# Differences in the detection of circulating Hsp90 alpha between patients with atopic dermatitis and dermatitis herpetiformis

**DOI:** 10.3389/fmed.2023.1327144

**Published:** 2024-01-05

**Authors:** Krzysztof Sitko, Sarolta Kárpáti, Grzegorz Węgrzyn, Grzegorz Mincewicz, Magdalena Trzeciak, Michael Kasperkiewicz, Stefan Tukaj

**Affiliations:** ^1^Department of Molecular Biology, Faculty of Biology, University of Gdańsk, Gdańsk, Poland; ^2^Department of Dermatology, Venereology and Dermatooncology, Semmelweis University, Budapest, Hungary; ^3^Department of Dermatology, Venereology and Allergology, Medical University of Gdańsk, Gdańsk, Poland; ^4^Department of Dermatology, Keck School of Medicine, University of Southern California, Los Angeles, CA, United States

**Keywords:** autoimmune diseases, atopic dermatitis (AD), dermatitis herpetiformis (DH), heat shock protein 90 (Hsp90), COVID-19

## Abstract

Heat shock protein 90 alpha (Hsp90α) is one of the key intra- and extracellular chaperones responsible for the biological activity of various signaling molecules that are involved in (auto)immune-mediated inflammatory diseases. Recent epidemiologic data suggest that patients with atopic dermatitis (AD) are at risk for several autoimmune diseases, including dermatitis herpetiformis (DH), an extraintestinal manifestation of celiac disease (CD). In addition, pruritic diseases such as AD may be confused clinically with DH. In this study, we aimed to determine the role of circulating Hsp90α in patients with AD in relation to patients with DH, CD, and healthy controls. Using an enzyme-linked immunosorbent assay, levels of circulating Hsp90α were determined in serum samples derived from patients with AD (*n* = 31), DH (*n* = 26), CD (*n* = 15), and healthy controls (*n* = 55). Although serum concentrations of Hsp90α were similar between patients with DH, CD, and healthy controls, we found that serum levels of Hsp90α were significantly higher (mean value of 5.08-fold; *p* < 0.0001) in patients with AD when compared to patients with DH. A cutoff value calculated as 2 × standard deviation above the mean concentration of Hsp90α in DH patients revealed that 83.9% (26/31) of AD patients were Hsp90α positive, whereas none of the DH patients (0/26) displayed such a positivity. This preliminary study suggests a distinct role for extracellular Hsp90α in the pathogenesis of AD compared to DH and its potential use in distinguishing AD from DH. Nevertheless, the potential role of the evaluation of extracellular Hsp90α for distinguishing between AD and DH is at present speculative and requires further and careful observations.

## 1 Introduction

The heat shock protein 90 (Hsp90) family consists of four members, i.e., the two cytosolic isoforms Hsp90α and Hsp90β, and grp94/gp96 and TRAP-1 isoforms, localized in the endoplasmic reticulum and mitochondrion, respectively. Hsp90α is an essential molecular chaperone responsible for the biological activity of hundreds of signaling molecules (e.g., mitogen-activated protein kinase (MAPK) or Janus kinase/signal transducer and activator of transcription (JAK/STAT)) and transcription factors (e.g., nuclear factor-kappa B (NF-κB)) that are involved in the pathophysiology of immune-mediated diseases, including non-communicable inflammatory skin diseases (1). In fact, Hsp90α can be passively or actively secreted into the extracellular space to promote cell motility and mediate wound healing and tumor metastasis (2). We have previously found that the sera of patients with dermatitis herpetiformis (DH) or atopic dermatitis (AD) contained significantly elevated titers of anti-Hsp90 IgG or anti-Hsp90 IgE, respectively (3, 4). In addition, circulating levels of Hsp90α were found to be significantly elevated in AD patients when compared to healthy controls and positively associated with the severity of AD assessed by Scoring Atopic Dermatitis (SCORAD) (4). The role of circulating Hsp90α in DH, however, has not been studied so far.

AD is one of the most common chronic inflammatory skin diseases, characterized by intense itching and relapsing skin lesions (5). In contrast, DH, an extraintestinal manifestation of celiac disease (CD) presenting similarly with intensely pruritic skin lesions, is a relatively rare autoimmune blistering skin disease (6). Due to the wide range of heterogeneity, there is no specific biomarker for AD diagnosis, and it is made based mainly on clinical features (7). On the other hand, determination of levels of anti-epidermal transglutaminase (eTG/TG3) IgA autoantibodies was shown to be a primary diagnostic strategy, with estimating levels of anti-tissue transglutaminase (tTG/TG2) and other CD-associated autoantibodies used to support the diagnosis or activity monitoring of DH (8).

Recent epidemiologic data suggest that patients with AD are at risk for several autoimmune diseases, including DH (9). In addition, DH can be misdiagnosed as AD due to a similar clinical phenotype or the general higher prevalence of AD (8). Therefore, there are still unmet needs to find more convenient, non-invasive, and reliable biomarkers that would both allow for a more efficient differentiation of these skin diseases and facilitate to the proposal of appropriate or personalized therapy.

In this study, we aimed to determine the role of circulating Hsp90α in patients with AD in relation to patients with DH, CD, and healthy controls by analyzing its serum levels and associations with disease serological markers.

## 2 Materials and methods

### 2.1 Patients

Sera from 31 patients with active atopic dermatitis (mean age: 27.77 ± 13.59 years; gender: 15 males and 16 females) with an average SCORAD index of 51.79 ± 18.99 and elevated total circulating IgE antibodies (mean 1159.06 ± 772.50 U/mL) without concomitant DH were included in this study. According to the SCORAD index, the mild form of AD was recorded in 3 patients, the moderate form in 13 patients, and the severe form in 13 patients. In two cases, the SCORAD index was not defined. Basic therapy including emollients and topical anti-inflammatory first-line drugs such as glucocorticoids or calcineurin inhibitors were used by most of AD patients prior to recruitment. AD patients using systemic treatment (e.g., phototherapy, systemic corticosteroids, cyclosporine, methotrexate, azathioprine, JAK inhibitors, and biologics) were excluded from the study. Sera from 26 patients with active and untreated DH (mean age 35.42 ± 14.93 years; gender: 12 females and 14 males) and elevated circulating anti-eTG autoantibodies (mean 81.81 ± 87.19 U/mL) and anti-tTG autoantibodies (mean 108.80 ± 103.54 U/mL) were included in this study. Sera from 15 patients with active and untreated CD (mean age: 35.27 ± 13.84 years; gender: 12 females and 3 males) and elevated circulating anti-tTG autoantibodies (mean 129.27 ± 120.48 U/mL) and anti-eTG autoantibodies (80.94 ± 86.79) without concomitant DH were included in this study. In this study, three types of controls without a history of immunological, allergic, or skin disorders were used. Sera from (i) 26 anti-SARS-CoV-2 IgG-positive volunteers (mean age: 44.92 ± 13.82; gender: 4 males and 22 females) without a history of COVID-19 who received two doses of the mRNA anti-COVID-19 vaccine encoding the viral spike protein (Pfizer-BioNTech COVID-19 vaccine), (ii) 15 unvaccinated COVID-19 convalescents (anti-SARS-CoV-2 IgG-positive; mean age: 30.40 ± 13.00; gender: 10 males and 5 females), and (iii) 55 healthy controls (anti-SARS-CoV-2 IgG-negative; mean age: 27.91 ± 3.21; gender: 23 males and 32 females) have been included in this study. The collection of sera from patients with AD, DH, and CD was conducted before the outbreak of the COVID-19 pandemic. None of the patients with AD, DH, or CD were COVID-19 vaccinated. All patients and healthy volunteers that were recruited in this study were Caucasian.

### 2.2 Detection of circulating Hsp90α

Hsp90α levels were evaluated in the serum using a commercially available HSP90α (human) ELISA kit (Enzo Life Science), following the manufacturer’s instructions.

### 2.3 Detection of circulating anti-SARS-CoV-2 antibodies

The presence of circulating anti-SARS-CoV-2 antibodies directed to the S1 subunit of the spike protein or nucleocapsid protein (NCP) was evaluated using a commercially available anti-SARS-CoV-2 ELISA (IgG) kit (EUROIMMUN) or anti-SARS-CoV-2 NCP ELISA (IgG) kit (EUROIMMUN), respectively, following the manufacturer’s instructions.

### 2.4 Statistical analysis

Statistical analyses were performed using the GraphPad Prism software (San Diego, CA). Data were analyzed by one-way ANOVA test with Tukey’s multiple comparison test. For correlation analysis, the Spearman rank correlation coefficient test was used. Fisher’s exact test was used to determine the significance of associations between groups. The *p*-values below 0.05 were considered significant.

## 3 Results

**Figure 1:**
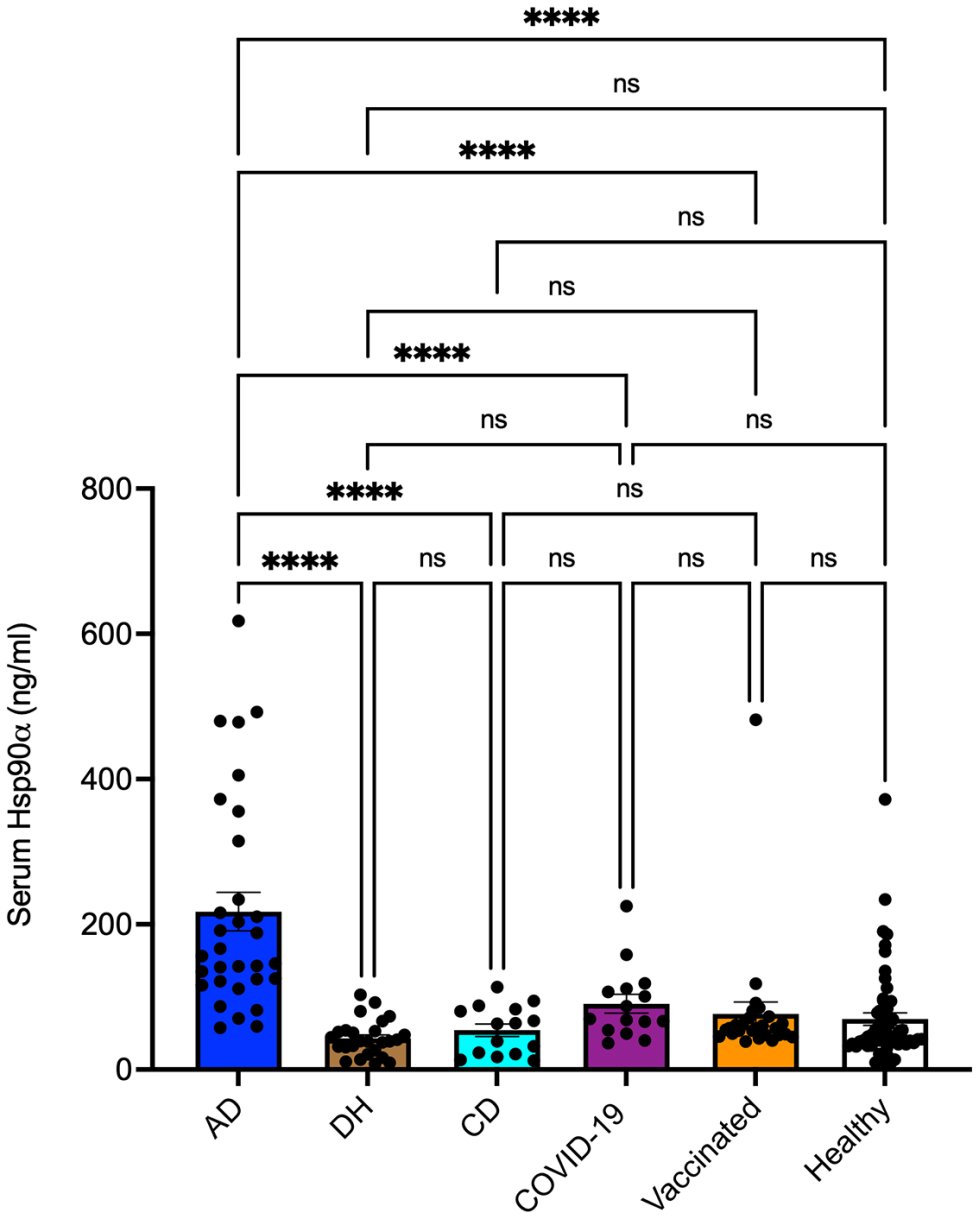
Levels of Hsp90α in sera of patients with atopic dermatitis (AD; *n* = 31), dermatitis herpetiformis (DH; *n* = 26), celiac diseases (CD; *n* = 15), COVID-19 convalescence status (COVID-19; *n* = 15), anti-COVID-19 vaccination status (Vaccinated; *n* = 26), and healthy controls (Healthy; *n* = 55). Serum levels of Hsp90α were assessed by an enzyme-linked immunosorbent assay. The dots and bars indicate individual and mean values in each group, respectively. Data were analyzed by a one-way ANOVA test with Tukey’s multiple comparison test. **** *p* < 0.0001. The *p*-values below 0.05 were considered significant. ns, not significant.

Although serum concentrations of Hsp90α in patients with DH (*n* = 26) or CD (*n* = 15) and healthy controls (*n* = 55) remained unchanged, we found that serum levels of Hsp90α were significantly higher (5.08-fold; *p* < 0.0001) in patients with AD (*n* = 31) when compared to patients with DH (Figure 1). The cutoff value calculated as 2 x standard deviation (SD) above the mean concentration of Hsp90α in DH patients revealed that 83.9% (26/31) of AD patients were Hsp90α positive, whereas none of the DH patients (0/26) displayed such positivity. As analyzed by the Fisher’s exact test, these differences were found to be statistically significant (*p* < 0.0001). Neither anti-eTG IgA nor anti-tTG IgA autoantibodies present in the serum of both DH and CD patients correlated with circulating Hsp90α levels (data not shown). Seropositivity to SARS-CoV-2, due to infection or immunization, did not impact Hsp90α concentration measurements, as both groups of seropositive patients had comparable serum Hsp90α levels when compared to seronegative donors (Figure 1).

## 4 Discussion

Epidemiologic data suggest that patients with AD are at risk for several autoimmune diseases, including DH (9). Indeed, approximately 20% of Hungarian and Finnish children with DH suffer from AD (10). Furthermore, due to the similar clinical phenotype of a severely itchy skin rash or the higher prevalence of AD in the general population, DH may mimic AD and be misdiagnosed (8). Detecting circulating IgA autoantibodies to eTG or tTG, however, may help in diagnosing DH. Ultimately, DH can be distinguished from AD by the detection of IgA deposits in the dermal papillae or at the dermal-epidermal junction (6, 11). Nevertheless, the mean delay in the diagnosis of DH has been reported to be as long as 3.2 years (12), suggesting a still imperfect diagnostic scheme that should certainly be significantly shortened.

Our previous observations showed that sera from patients with DH, CD, and AD contained significantly increased titers of anti-Hsp90 IgG, anti-Hsp90 IgA, and anti-Hsp90 IgE, respectively (3, 4, 13). Interestingly, circulating levels of Hsp90α were significantly increased in AD patients compared with healthy controls and positively correlated with AD severity (4). In this study, we found that serum levels of Hsp90α were significantly higher in patients with AD when compared to patients with DH and CD or healthy controls. This preliminary outcome suggests a distinct role for extracellular Hsp90α in the pathogenesis of AD compared to DH and its potential use as a biomarker in distinguishing AD from DH. It should also be considered that several studies on infectious, oncological, and (auto)immune diseases have already demonstrated the potential of Hsp90 as a biomarker of human diseases (14). Considering the recent COVID-19 pandemic, it is worth noting that seropositivity to SARS-CoV-2, due to infection or immunization, did not impact Hsp90α concentration measurements, as both groups of seropositive patients had comparable serum Hsp90α levels when compared to seronegative donors.

Distinct pathophysiological roles of extracellular Hsp90α in the development of AD, DH, and CD are indicated by different statistical associations found between circulating levels of Hsp90α or autoantibodies to Hsp90 and disease activity or conventional serological biomarkers. For instance, we have previously found that higher titers of anti-Hsp90 IgG are positively correlated with serum levels of anti-eTG or anti-tTG IgA autoantibodies, as well as disease activity in DH patients (3). Likewise, higher levels of anti-Hsp90 IgA were found to be positively associated with anti-tTG IgA autoantibody levels in patients with CD (13). In contrast, an association between circulating anti-Hsp90 IgE and disease activity (SCORAD) was not recorded in patients with AD (4). In this study, neither anti-eTG IgA nor anti-tTG IgA autoantibodies present in the serum of both DH and CD patients correlated with circulating Hsp90 levels.

Our study has some limitations. For instance, basic therapy, including emollients and topical anti-inflammatory first-line drugs such as glucocorticoids or calcineurin inhibitors, was used by most AD patients prior to recruitment. In contrast, only patients with untreated DH were included. On the other hand, AD patients using systemic treatment (e.g., phototherapy, systemic corticosteroids, cyclosporine, methotrexate, azathioprine, JAK inhibitors, and biologics) that could potentially influence the Hsp90 content in the blood were excluded from the study.

## 5 Conclusion

Our results suggest a distinct role for extracellular Hsp90α in the pathogenesis of AD and DH and its potential use in distinguishing AD from DH. While the determination of anti-eTG and anti-tTG autoantibodies was shown to be a primary strategy used to support the diagnosis or activity monitoring of DH (specificity >90–95%) (8), we believe that the assessment of Hsp90α levels may help the physician distinguish DH from AD in those cases where serological results regarding the level of anti-eTG and anti-tTG autoantibodies are equivocal with a simultaneous low level of circulating Hsp90α. Nevertheless, further research using larger cohorts is necessary to standardize the Hsp90α detection method and to determine its accuracy, sensitivity, and specificity. Additionally, to better understand the role of Hsp90α in the development of AD and DH, future analysis of the expression of this protein in skin biopsies from patients should be considered.

## Data availability statement

The raw data supporting the conclusions of this article will be made available by the authors, without undue reservation.

## Ethics statement

The studies involving humans were approved by ethics committees of the Semmelweis University, Medical University of Gdańsk, and a bioethics committee at the regional medical chamber in Gdańsk, Poland. The studies were conducted in accordance with the local legislation and institutional requirements. The participants provided their written informed consent to participate in this study.

## Author contributions

KS: Data curation, Formal analysis, Investigation, Methodology, Software, Validation, Visualization, Writing – review & editing. SK: Writing – review & editing. GW: Writing – review & editing. GM: Writing – review & editing. MT: Writing – review & editing. MK: Writing – review & editing. ST: Conceptualization, Data curation, Formal analysis, Funding acquisition, Investigation, Methodology, Project administration, Resources, Software, Supervision, Validation, Visualization, Writing – original draft.
